# Multiple endocrine neoplasia type 2B diagnosed after small intestinal volvulus with progressive megacolon in an adolescent

**DOI:** 10.1007/s12328-024-01979-y

**Published:** 2024-05-16

**Authors:** Yusuke Sakai, Yoshiko Nakayama, Shingo Kurasawa, Tomomitsu Sado, Sawako Kato, Nao Hidaka, Shigeru Takamizawa, Katsumi Yoshizawa, Koichiro Yoshimaru, Tomoaki Taguchi

**Affiliations:** 1 Department of Pediatrics, Shinshu University School of Medicine, 3-1-1 Asahi, Matsumoto, Nagano Japan; 2 Department of Surgery, Nagano Children’s Hospital, 3100 Toyoshina, Azumino, Nagano Japan; 3 Department of Pediatric Surgery, Reproductive and Developmental Medicine, Faculty of Medical Sciences, Kyushu University, Fukuoka, Japan; 4 Fukuoka College of Health Sciences, Fukuoka, Japan

**Keywords:** Multiple endocrine neoplasia type 2B, Constipation, Megacolon, Volvulus, Ganglioneuromatosis

## Abstract

Multiple endocrine neoplasia type 2B is a rare autosomal dominant disease characterized by the presence of medullary thyroid carcinoma, pheochromocytoma, Marfan-like fatigue, a peculiar face with thickening of the lips, mucosal neuromas on the lips and tongue, and gastrointestinal phenomena. Most patients harbor pathological variants of the *RET* gene. Herein, we present the first case of a 14 year-old boy who experienced small intestinal volvulus along with a megacolon, and he was diagnosed with multiple endocrine neoplasia type 2B. The patient complained of constipation since he was 2 years old and slowly progressive abdominal distension at school age. At 14 years of age, he presented with remarkable megacolon mimicking Hirschsprung’s disease and complicated with small intestinal volvulus. The volvulus was successfully repaired, and the particularly dilated transverse colon was resected following a rectal biopsy. Histopathological evaluation of the resected transverse colon revealed to be compatible with ganglioneuromatosis. After emergency surgery, the patient was diagnosed with multiple endocrine neoplasia type 2B with medullary thyroid carcinoma, and a de novo variant of *RET* was confirmed*.* Gastroenterologists should consider it when treating patients with constipation, especially those with megacolon. Therefore, timely diagnosis may lead to appropriate treatment of medullary thyroid carcinoma and improve mortality.

## Introduction

Multiple endocrine neoplasia (MEN) is a rare autosomal dominantly inherited cancer predisposition syndrome [1] classified into MEN types 1 and 2. Multiple endocrine neoplasia type 2 includes the following phenotypes: MEN type 2A (MEN 2A), familial medullary thyroid carcinoma (MTC), and MEN type 2B (MEN 2B) [2]. Multiple endocrine neoplasia type 2A accounts for > 80% of MEN type 2 cases, whereas MEN 2B accounts for 5% of cases [3, 4]. All phenotypes involve a high risk of developing MTC. Approximately 95% of MEN 2B patients have pathological variants in the *RE*arranged during *T*ransfection (*RET*) gene (10q11.2) p.Met918Thr in exon 16 [2, 4]. However, approximately 75% of MEN 2B cases are sporadic and affect patients that have a de novo pathological variant of *RET*, whereas 25% of cases occur in families with previous or current manifestations of MEN 2B [4]. In 2015, the American Thyroid Association guidelines on MTC management recommended that patients with MEN 2B and the *RET* codon M918T variant be categorized as “highest risk” of MTC [4].

Additional features of MEN 2B include pheochromocytoma, Marfan-like fatigue, a peculiar face with thickening of the lips, mucosal neuromas on the lips and tongue, tearless cry (alacrima), and ganglioneuromatosis of the gastrointestinal tract [2, 5]. Gastrointestinal symptoms or abnormalities, which include constipation, diarrhea, and megacolon, were frequently observed in patients with MEN 2B from infancy to adulthood before the diagnosis of MEN 2B [5–8]. Alimentary tract ganglioneuromatosis is a well-known histopathological phenomenon in patients with MEN 2B [5, 6, 8]. However, it is difficult for clinicians to diagnose extremely rare disease in children or adolescents with undiagnosed MEN 2B who complain of common gastrointestinal symptoms.

We encountered a patient who was presented with constipation and slowly progressive abdominal distension at school age. This was complicated by small intestinal volvulus with remarkable megacolon mimicking Hirschsprung’s disease, which was diagnosed with sporadic MEN 2B. With informed consent from the patient and guardians, we present this case report along with a literature review.

### Case report

**Fig. 1:**
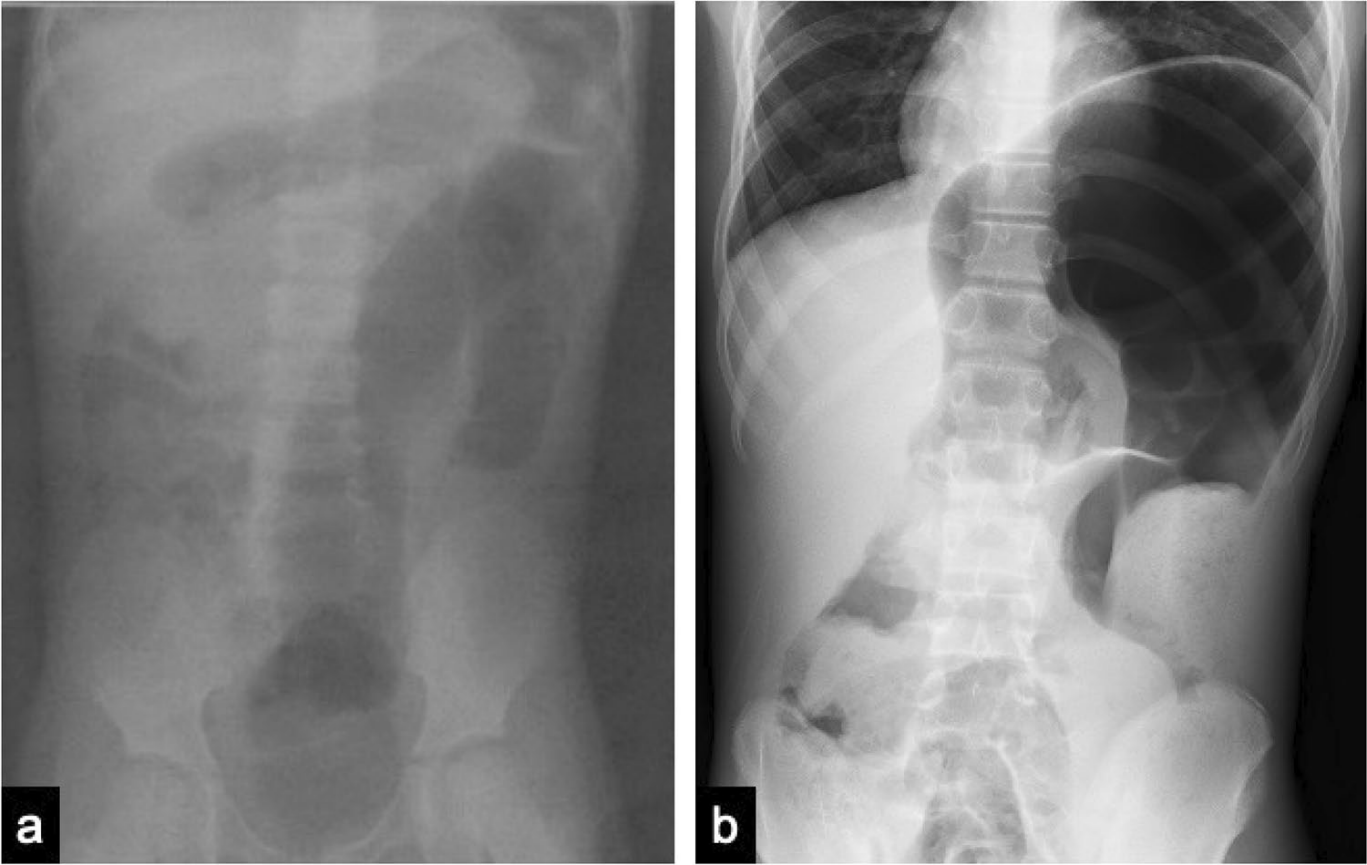
Composite figure showing X-ray of the patient with multiple endocrine neoplasia type 2B. **a** Plain abdominal X-ray at first visit (6 year-old) showing mild colon dilation. **b** Plain abdominal X-ray at 14 years of age showing significantly dilated colon

**Fig. 2:**
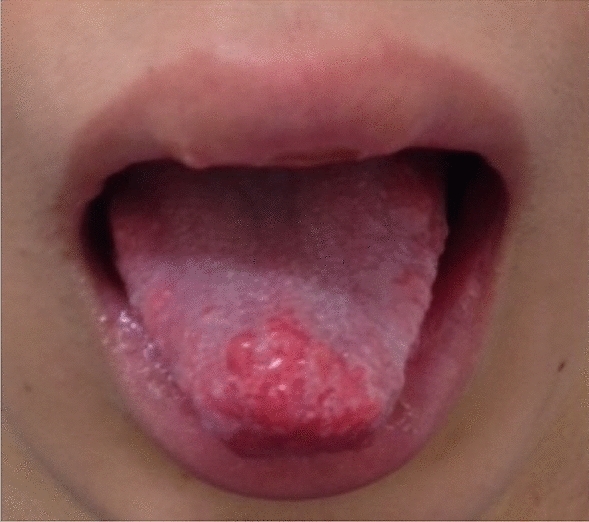
Mucosal neuromas on the tongue

A 14 year-old boy was presented to our hospital with a one-year history of abdominal bloating and constipation. The patient had no family history of MEN 2B, thyroid cancer, or pheochromocytoma. His perinatal period was normal and no delay in passage of meconium as a neonate or complaints of constipation during infancy. When he was 2 years old, megacystis was suspected; however, a pediatric urologist later ruled out a megacystis and did not require any treatment or follow-up. At 6 years of age, he visited a hospital with a complaint of constipation and was noted to have mild colonic dilatation (Fig. 1a). A rectal biopsy was suggested to rule out Hirschsprung’s disease; however, the patient and parents declined the biopsy because his defecation improved with enemas. When he was 7 years old, he was presented with a tongue tubercle (Fig. 2), which was histopathologically diagnosed as a submucosal neuroma. When he was 14 years old, abdominal distension developed, and he was referred to our hospital. At first consultation, his height was 165.2 cm, weight was 43.4 kg, and body mass index was 15.9 (three percentiles of age). Abdominal radiography revealed significant intestinal dilatation (Fig. 1b). On barium enema examination, the ascending and transverse colons were dilated, but there were no remarkable caliber changes. We suspected an allied disorder of Hirschsprung’s disease (ADHD) and scheduled an intestinal biopsy.

**Fig. 3:**
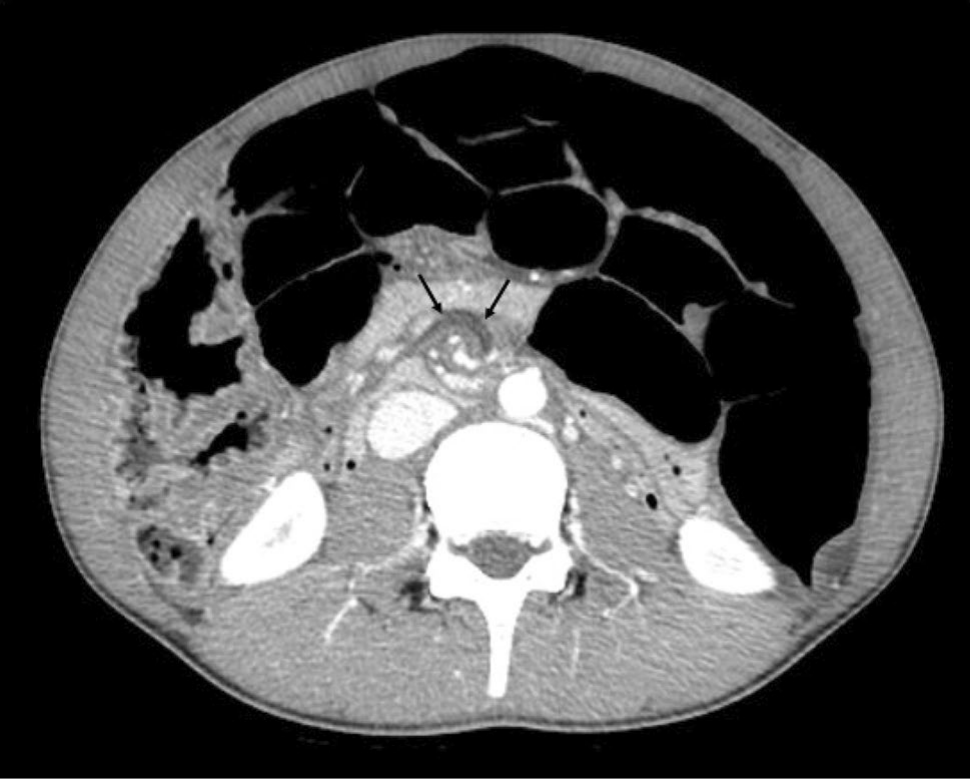
Enhanced computed tomography showing colonic dilation, narrowing of superior mesenteric vein, and whirlpool sign (arrows) of mesentery that suggests small intestinal volvulus

**Fig. 4:**
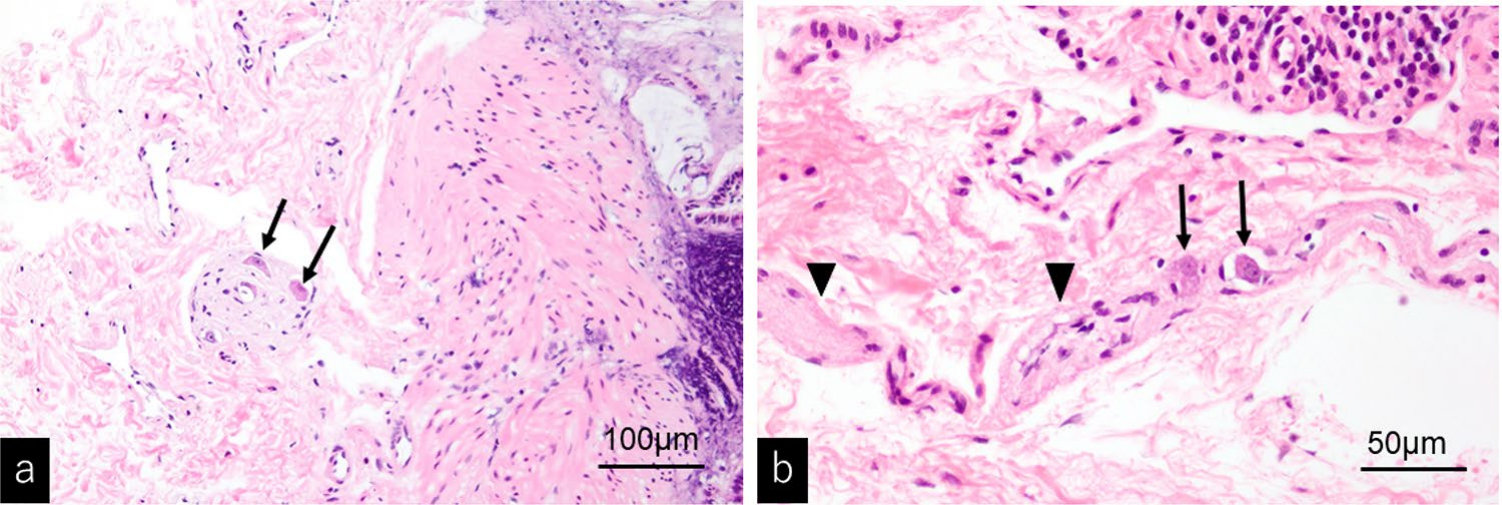
Histopathological features of the rectal biopsy (H&E staining). **a** Low power magnification. **b** High power magnification. Several ganglion cells (arrows) with proliferation of thick nerve bundles (arrowheads) are identified in the submucosal layer. Some of them showed the tumor like appearance **a**. Hirschsprung’s disease was excluded

**Fig. 5:**
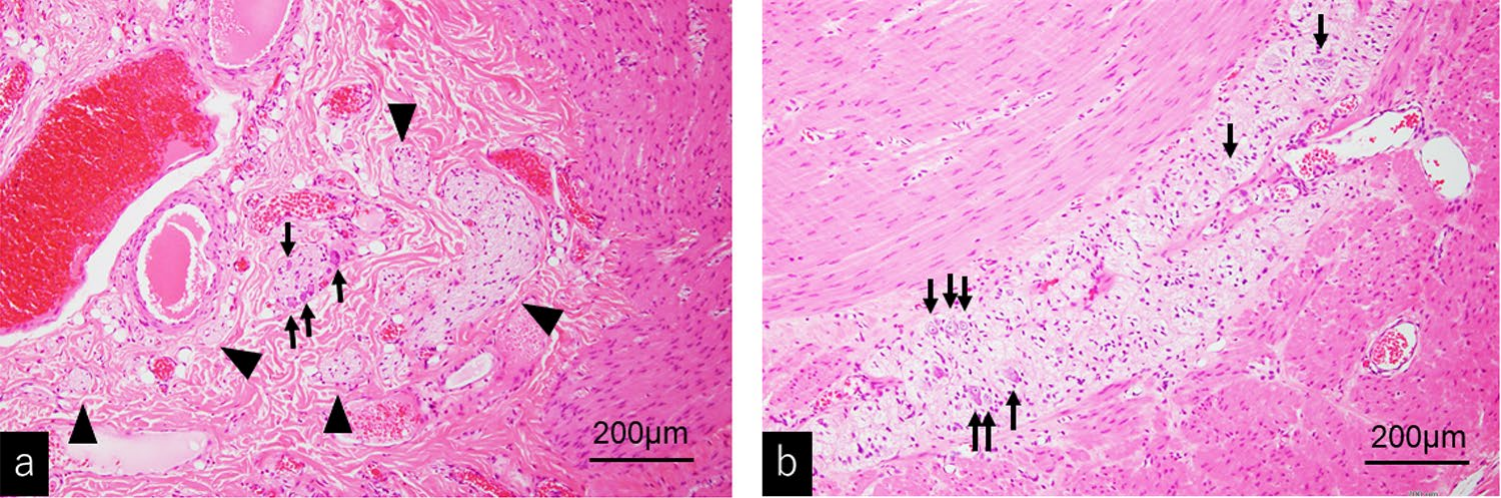
Histopathological features of resected transverse colon (H&E staining). **a** The proliferation of ganglion cells (arrow) and thick nerve bundles (arrowheads) are frequently seen in the submucosal layer. **b** Giant plexuses containing numerous ganglion cells (arrows), glial tissue, and nerve fibers occupied in the intermuscular zone. Some of them showed tumor-like appearance. These findings are compatible with those of ganglioneuromatosis

**Fig. 6:**
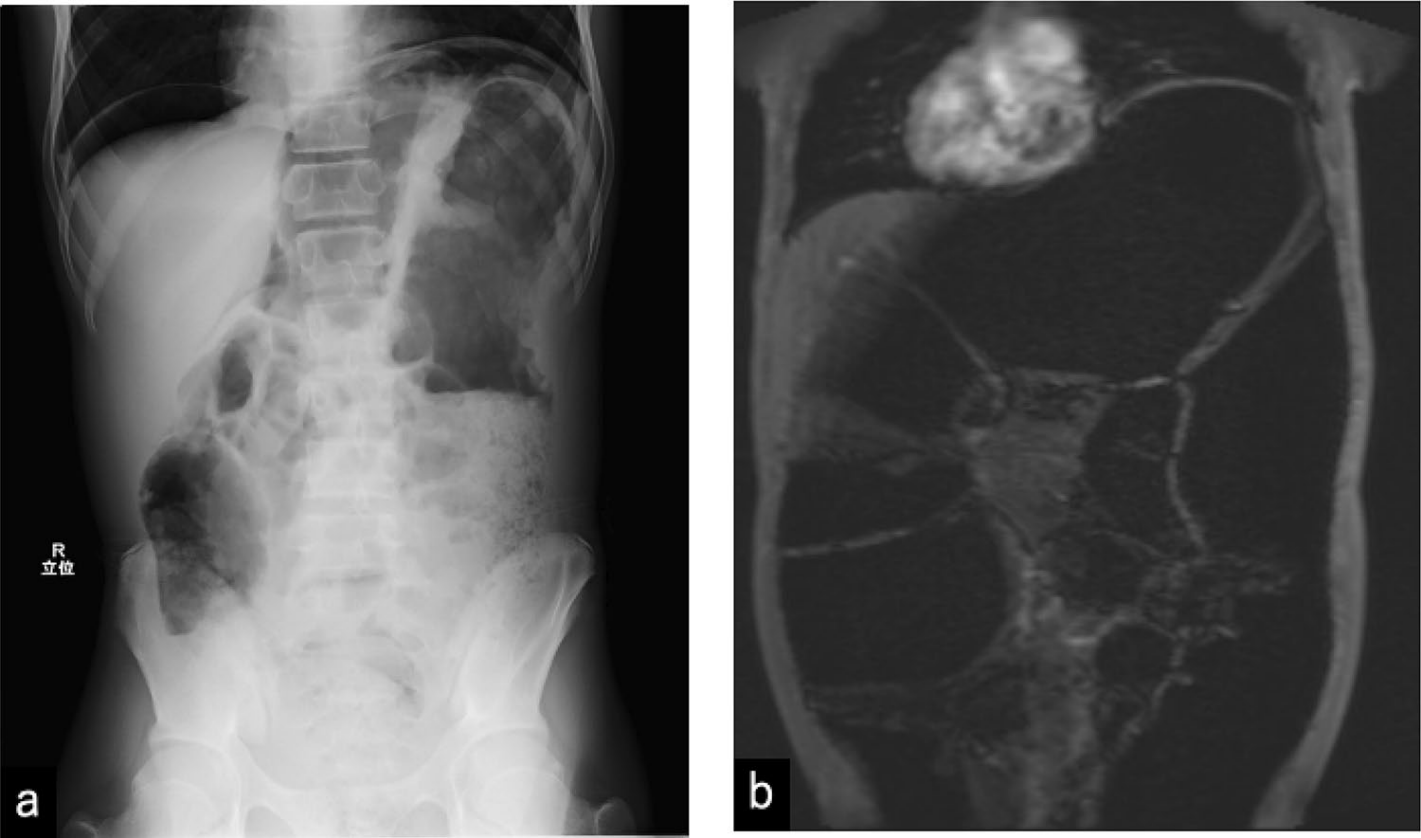
Composite figure showing radiological features after operation. **a** Plain abdominal X-ray at post-operation day six showing mild colonic dilation at the age of 14 years. **b** Abdominal magnetic resonance imaging of the anterior segment showing prominent diffuse megacolon at the age of 20 years.

Three months later, while waiting for a scheduled biopsy, he visited the emergency department of our hospital due to sudden bilious vomiting and severe abdominal pain. He had an anguished appearance, and his abdomen was significantly distended. There was tenderness to the left of the umbilicus, but no sign of peritoneal irritation. The results of blood tests are shown in Table 1. Emergency enhanced computed tomography revealed colonic dilation, narrowing of the superior mesenteric vein, and a whirlpool sign (arrows) in the mesentery (Fig. 3). Small intestinal volvulus was diagnosed, and the patient underwent emergency surgery. Intraoperatively, the small intestine was twisted by 360°; however, there was no sign of intestinal ischemia, and the volvulus was successfully repaired. The colon was diffusely dilated, which may have induced volvuli in the small intestine. The transverse colon, which was particularly dilated up to 8 cm in diameter and 17 cm in length, was resected, and rectal biopsy was performed. The histopathological findings of the rectal biopsy showed ganglion cells (arrows) (Fig. 4a) and the numerous thick neuronal bundles (arrowheads) (Fig. 4b) in the submucosal layer with hematoxylin and eosin (H&E) staining and acetylcholinesterase (AchE) staining. Therefore, Hirschsprung’s disease was excluded. Some of the submucosal plexuses showed tumor-like appearance (Fig. 4a). The histopathological findings of the resected dilated transverse colon revealed the proliferation of ganglion cells (arrow), thick nerve bundles (arrowheads) in the submucosal layer (Fig. 5a). We also found the giant plexuses containing many ganglion cells (arrows), many glial cells, and nerve fibers in the intramuscular layer (Fig. 5b). These findings were compatible with those of ganglioneuromatosis. The patient was discharged on day seven after admission without postoperative complications. Abdominal radiography revealed mild dilation of the colon at the time of discharge (Fig. 6a).

**Table 1:** Results of blood tests at the time of emergency surgery

WBC	7730/μL	TP	7.2 g/dL	Na	140 mmol/L
SEG	28%	Alb	4.8 g/dL	K	3.7 mmol/L
BND	0%	BUN	12.6 mg/dL	Cl	106 mmol/L
LYM	60%	Cre	0.58 mg/dL	Glu	109 mg/dL
EOS	8%	AST	21 U/L	Venus blood gas	
MON	3%	ALT	12 U/L	pH	7.351
BAS	1%	γ-GTP	12 U/L	pCO2	47.2 mmHg
RBC	5.23 × 10^6^/μL	LDH	200 U/L	pO2	42.4 mmHg
Hb	15.3 g/dL	CK	129 U/L	HCO3	25.5 mEq/L
PLT	23.5 × 10^4^/μL	Amy	40 U/L	BE	− 0.2 mEq/L
		CRP	0.01 mg/dL	Lac	20 mg/dL

After the surgery for volvulus, we confirmed hypercalcitoninemia on blood tests and diagnosed the patient with MTC, which was confirmed by ultrasonography, magnetic resonance imaging, and biopsy. His face was peculiar with thick lips and a Marfan-like appearance. Based on the clinical manifestations of MTC, mucosal neuromas on the tongue, ganglioneuromatosis of the gastrointestinal tract, Marfan-like appearance, and a peculiar face, we clinically diagnosed the patient with MEN 2B and confirmed a germline pathological variant of *RET* gene, p.Met918Thr (c.2753T > C). After thyroidectomy and parathyroid gland autoplantation, the patient was administered levothyroxine sodium hydrate. His thyroid stimulating hormone and free thyroxine levels remained normal. No MTC metastases or pheochromocytoma were observed under careful surveillance His thyroid stimulating hormone and free thyroxine levels remained normal. No MTC metastases or pheochromocytoma were observed under careful surveillance based on the blood tests (Table 2) and imaging studies as appropriate. He had spontaneous defecation once a day and was followed-up for seven years without re-operation, although the megacolon is gradually exacerbated (Fig. 6b). Dimethicone, daikenchuto, and probiotics were prescribed to reduce flatulence, and laxatives were not prescribed.

**Table 2:** Results of recent blood tests

WBC	5,370/μL	TP	8.8 g/dL	Na	140 mmol/L
SEG	50%	Alb	4.3 g/dL	K	3.7 mmol/L
BND	0%	BUN	10.2 mg/dL	Cl	106 mmol/L
LYM	40%	Cre	0.76 mg/dL	FT-3	3.22 pg/mL
EOS	3%	AST	21 U/L	FT-4	2.03 ng/dL
MON	6%	ALT	15 U/L	TSH	2.75 μIU/mL
BAS	1%	γ-GTP	18 U/L	CEA	2.7 ng/mL
RBC	4.83 × 10^6^/μL	LDH	158 U/L	calcitonin	93.8 pg/mL
Hb	14.4 g/dL	Ca	8.8 mg/dL	metanephrine	142 pg/mL
PLT	20.8 × 10^4^/μL	P	5.0 mg/dL	normetanephrine	117 pg/mL

## Discussion

Multiple endocrine neoplasia 2B is a rare autosomal dominant disease characterized by the presence of MTC, pheochromocytoma, and a peculiar face [2]. Approximately 95% of all individuals with MEN 2B have a pathological variant in the tyrosine kinase domain of RET proto-oncogene at codon 918 in exon 16, which substitutes a threonine for methionine [2]. The estimated incidence of MEN 2B was no fewer than 1.4 per million live births per year between 1991 and 2000 [9]. About 75% of patients with typical MEN 2B have a de novo germline pathological variants of *RET* [4, 5, 10]. Among patients without family history of MEN 2B, the mean age at diagnosis was 14.2 years (range, 1–31 years), and all the patients had complications with MTC at diagnosis, and 33% died from MTC [11]. Moreover, Bracuckhoff et al. reported that severe intestinal manifestations were predominantly observed in patients with early onset MTC. These findings reflect the difficulty in the timely diagnosis of the syndrome in patients harboring a de novo pathological variant of *RET*. The present case was also a sporadic case with phenotypically normal parents, and it required a long period to diagnose MEN 2B, which was complicated by MTC. The authors emphasize that MEN 2B is an important disease that should not be forgotten as an organic disease with constipation, abdominal distention, and megacolon. This may help clinicians pay attention to Marfan-like fatigue, a peculiar facial appearance with thickening of the lips, and mucosal neuromas of the lips and tongue.

The importance of the alimentary tract components of MEN 2B was reported by Carney et al. in 1976 [8]. The frequency of gastrointestinal symptoms or abnormalities were observed in 14 of 16 patients (88%), which include constipation in 10 patients, diarrhea in three, and megacolon in five. Brauckhoff et al. reported that 74% (14/19 cases) of sporadic MEN 2B patients had intestinal dysfunction (constipation and/or diarrhea) before MEN 2B diagnosis [11]. Recently, Nagaoka et al. reported four Japanese cases of MEN 2B in both children and adults. Three of the four patients had gastrointestinal manifestations, one of whom had intestinal malrotation and megacolon at age 14 years of age, and the other had chronic constipation and flatulence since infancy [12]. According to previous reports, gastrointestinal symptoms in patients with MEN 2B included constipation (73–79%) [5, 13, 14], diarrhea (29–62%) [13, 14], and flatulence (79%) [14]. Moreover, the onset of gastrointestinal symptoms occurred during infancy or early childhood in 73% of patients [13]. Gastrointestinal manifestations offer the most important window of opportunity for early detection of MEN 2B. In the present case, we suspect that constipation at the age of 6 years was caused by mild loss of intestinal tone, and abdominal bloating and constipation at the age of 14 years was the result of megacolon.

Based on the radiographic findings, the megacolon was reported among 29–63% cases and considered a typical feature of MEN 2B [8, 13, 15]. Gibbons et al. reported that megacolon was diagnosed during infancy to elderly age among MEN 2B patients [15]. The ganglioneuromatosis could lead to loss of bowel tone, distension, segmental dilation and, ultimately megacolon [15]. In patients with megacolon, it is crucial to differentiate it from Hirschsprung’s disease [15, 16]. Hirschsprung's disease is characterized by a transit disorder of intestinal content, delayed meconium excretion, abdominal distention, bilious vomiting, constipation, and intestinal dilatation (megacolon) at the proximal side, resulting from dysperistalsis and a lack of a recto-anal reflex caused by aganglionosis of the intestinal tract at the distal side [17]. Transanal rectal biopsy has been proposed as a valid procedure to differentiate Hirschsprung disease from MEN 2B by detecting the presence of ganglion cells in the rectum [13, 18, 19]. It is known that 10–25% of MEN 2A cases with the *RET* gene pathological variants in codons 609, 611, 618, or 620 co-occur with Hirschsprung disease [7, 20]. However, few cases are complicated by Hirschsprung’s disease in MEN 2B [13].

The pathophysiological feature of the intestinal tract in MEN 2B was known as transmural intestinal ganglioneuromatosis [8, 11, 13, 21]. Ganglioneuromas are characterized by an increased number of ganglion cells, supportive cells, and nerve fibers in all bowel wall layers. Ganglioneuromas can lead to the loss of bowel tone, distension, segmental dilation, and ultimately megacolon [22]. This phenomenon is described by *RET* overactivation in MEN 2B, which lead to a large increase in intrinsic nerve fibers in the myenteric and submucosal ganglia, including the rectum [18, 21]. Ganglioneuromatosis extends from the lips to the rectum based on autopsy or surgical tissues [8]. In the present case, the histological findings were compatible with ganglioneuromatosis in the resected transverse colon. A review of previous reports evaluating the intestinal histopathology of MEN 2B showed that a small number of patients were diagnosed as intestinal neuronal dysplasia (IND) type B [13, 23]. In Japan, IND is currently classified as a subgroup of ADHD [17]. Additionally, ADHD is a disease group characterized by symptoms and signs similar to those of Hirschsprung’s disease, despite the presence of ganglionic cells in the rectum. Intestinal neuronal dysplasia type B accounts for up to 95% of IND cases. In terms of pathology, malformation of the intestinal parasympathetic nervous system is present, and AChE staining shows evidence of submucosal giant ganglia, hyperganglionosis, ectopic ganglia, and the growth of AChE-positive nerve fibers [17]. Histopathological differences between ganglioneuromatosis-like hyperplasia and IND type B remains controversial [24]. A reliable diagnosis of MEN 2B based on genetic testing and high-quality pathological diagnosis should be considered in a large number of cases. Moreover, histopathological evaluation of both the rectum and dilated colon is useful for the appropriate diagnosis of MEN 2B.

In MEN 2B patients with gastrointestinal symptoms, endocrinological screening and treatment are the first priority. Persistently elevated calcitonin levels induce diarrhea in patients with active MTC. Elevated secretion of catecholamines by pheochromocytomas may exacerbate reduced colonic tone, thereby leading to toxic megacolons [25]. After appropriate evaluation of endocrinopathies and neoplasms, most patients can manage their gastrointestinal symptoms conservatively with medication and dietary changes during childhood [7, 21]. However, gastrointestinal symptoms affect the patients’ quality of life [14]. More than one-third of patients with MEN 2B required gastrointestinal surgery, including colectomy for megacolon [7, 15, 21]. Surgery for megacolon is performed to reduce gastrointestinal symptoms and improve the quality of life. In children, some cases disappear symptoms after a single surgery. However, it is reported that patients who underwent colectomy in childhood needed re-operation in adulthood [7]. In the present case, the megacolon was exacerbated, and the dilated colon compressed the surrounding organs. Although the patient currently only has symptoms of flatulence and no symptoms of bowel obstruction, we are considering extended colon resection as a scheduled surgery, taking into account the invasiveness of the surgery and the possibility of re-operation in adulthood. Further evaluation of esophageal motility problems or evacuation disorders is required after colectomy [15]. To the best of our knowledge, this is the first report of a patient with MEN type 2B complicated by volvulus. A significantly dilated colon was suspected of occupying the abdominal cavity; thus, small intestinal volvulus was induced. Small intestinal volvulus is associated with the risk of intestinal ischemia and small intestinal resection.

In conclusion, we report the case of an adolescent with MEN 2B proceeding constipation and megacolon complicated by volvulus before the diagnosis of MEN 2B. Timely diagnosis may lead to appropriate treatment of MTC and improve mortality.

## Abbreviations

MEN: Multiple endocrine neoplasia
MEN 2A: Multiple endocrine neoplasia type 2A
MEN 2B: Multiple endocrine neoplasia type 2B
MTC: Medullary thyroid carcinoma
*RET*: *RE*arranged during *T*ransfection
ADHD: Allied disorders of Hirschsprung’s disease
H&E: Hematoxylin and eosin
AchE: Acetylcholinesterase
IND: Intestinal neuronal dysplasia
